# Prescription of Exercise Programs for Individuals with Autism Spectrum Disorder: Systematic Review

**DOI:** 10.1007/s10803-024-06566-1

**Published:** 2024-09-21

**Authors:** Sofia S. Ataíde, José P. Ferreira, Maria J. Campos

**Affiliations:** 1 University of Coimbra, Faculty of Sport Sciences and Physical Education, Coimbra University Stadium, Conímbriga Avenue, Pavilion 3, 3040-248 Coimbra, Portugal; 2 University of Coimbra, Sport and Physical Activity Research Unit (CIDAF, Uid/Dtp/04213/2020), Faculty of Sport Sciences and Physical Education, Coimbra, Portugal

**Keywords:** Physical exercise program, Resistance training, Heart rate, Strength capacity

## Abstract

Physical exercise offers health benefits for individuals with autism spectrum disorder (ASD). However, the literature on recommendations for exercise prescription is still limited. This study aims to identify the most beneficial type of exercise program and present the most effective guidelines for its prescription in individuals with ASD. A systematic review was carried out using the ERIC, Web of Science, PsycINFO, PubMed and Scopus databases in accordance with the PRISMA guidelines. Of a total of 1114 studies, 18 were considered eligible. Overall, the type of programs included aerobic exercise sessions with an average duration of 12 weeks, with 3 weekly sessions of 45 to 60 min. The assessment included the shuttle test protocol, the progressive cardiovascular endurance running protocol and the modified Bruce protocol. For the muscle strength exercises, the sessions lasted an average of 12 weeks, with 2 weekly sessions of 10 to 20 min, including 2 to 4 exercises, 1 to 3 sets, with 6 to 12 repetitions. The assessments included the handgrip strength test, the modified curve-up test and the push-up test. The programs had positive effects on cardiorespiratory capacity and hemodynamics, indicating that the benefits of training increase when both capacities are combined. This study provides useful guidelines for adapted sports coaches to prescribe exercise programs aimed at promoting quality of life in individuals with ASD.

## Introduction

According to the American Psychiatric Association (APA, 2022), autism spectrum disorder (ASD) is a complex category of neurobiological developmental disorders that are typically diagnosed in childhood and categorized into three dimensions: disturbance of verbal and nonverbal communication, social interaction and the presence of repetitive and stereotyped behaviors (Diagnostic and Statistical Manual of Mental Disorders, 2022).

In recent decades, the worldwide prevalence of ASD has been set at approximately 1% (Baird et al., 2011). Between 2009 and 2017, the National Health Interview Survey (NHIS) (Zablotsky et al., 2019) reported significant variation in the prevalence of ASD, from 1.12 to 2.49% (*p* < 0.01), i.e., an increase of 122.3% in eight years. According to the World Health Organization (WHO, 2020), the prevalence of ASD in Europe presents a wide range of estimates, reporting an average rate of 61.9/10,000 individuals, with a variation between 30.0 and 116.1/10,000 individuals (Elsabbagh et al., 2012). Recent results from the ASD in the European Union Program (ASDEU, 2018), point to an estimated average prevalence of 12.2/ 1000 individuals, i.e. one in 89 children between the ages of 7 and 9. Global estimates of ASD prevalence among different European countries range from 4.4 to 19.7/1000 children aged 7–9 (10th and 90th percentiles).

On the basis of epidemiological studies carried out in recent years, it is possible to state that the prevalence of ASD is increasing worldwide. There are several possible explanations for this increase, including greater awareness of the condition, the expansion of diagnostic criteria, more effective diagnostic tools and more detailed reporting (Zeidan et al., 2022). In this sense, managing the health and well-being of individuals with ASD is fundamental, but it is a complex process. The wide range of symptoms and comorbidities associated with this condition makes it very difficult to manage, as evidenced by the co-existence of multiple health problems. Sleep disorders, metabolic disorders, motor deficits, gastrointestinal problems and obesity are identified as the most common comorbidities in the ASD population (Jesner et al., 2007; Srisawasdi et al., 2017; Suárez-Manzano et al., 2024).

Individuals with ASD have significantly lower levels of physical exercise than their peers do (Jones et al., 2017; Rimmer & Rowland, 2008; Srinivasan et al., 2014). A recent study compared the levels of physical exercise in adults with ASD aged between 18 and 27 years with those in neurotypical adults in the same age group and revealed that, compared with 50% of the neurotypical group, only 10% of the participants with ASD exercised regularly (Hillier et al., 2020).

In this sense, the cumulative effect of primary symptoms, associated comorbidities, the continuous use of drugs and a sedentary lifestyle seem to make the population with ASD more vulnerable to body weight gain (Broder-Fingert et al., 2014), with more than half not performing the recommended amount of physical exercise (Carroll et al., 2014; Gawlik et al., 2018), i.e., 150 min of moderate to vigorous intensity per week, according to the guidelines of the American College of Sports Medicine (2022), resulting in a lower percentage of fat-free mass (González-Agüero et al., 2011), a lower resting metabolic rate and, consequently, lower energy expenditure (Hills et al., 2013; Polfuss et al., 2018). We are therefore dealing with a sedentary population characterized essentially by low caloric expenditure, which predicts low physical fitness in terms of cardiorespiratory and muscle strength (Salaun & Berthouze-Aranda, 2012).

Cardiorespiratory fitness is the ability to perform moderate- to high-intensity exercise, encompassing the main muscle groups, for long periods of time. It is a physiological marker of cardiovascular health that combines the respiratory, cardiovascular and musculoskeletal systems (Booth et al., 2012; Harber et al., 2017; Ruivo, 2018). Increasing the intensity of exercise requires the body to proportionally increase oxygen consumption, reaching a maximum level, defined as maximum oxygen consumption (VO^2^max) (Ruivo, 2018; Wilmore et al., 2001). According to Fleck and Kraemer (2017), muscle strength training aims to adapt skeletal muscles through overload, resulting in increased activity of glycolytic enzymes and adenosine triphosphate production with adaptations in the nervous system, leading to an increase in the number of motor units recruited. Individuals with ASD have lower levels of muscle strength than their peers with typical development (Golubović et al., 2012 and Borji et al., 2019), in which the patterns of fatigue and neuromuscular recovery are also lower (Zafeiridis et al., 2005).

Therefore, recent data have shown that the inclusion of physical exercise in intervention programs for individuals with ASD can have a potentially beneficial effect (Bremer et al., 2016). Several studies have shown that physical exercise has a positive influence on different symptoms and comorbidities, such as obesity and overweight problems (Dickinson & Place, 2014; Fragala-Pinkham et al., 2008); reduces motor deficits (Batey et al., 2014) and task execution time (Oriel et al., 2011); improves the psychopathological profile and cognitive function (Bremer et al., 2016; Tan et al., 2016); reduces stereotypes (Ferreira et al., 2019; Toscano et al., 2018) and aggressive behavior (Neely et al., 2015); and improves socioemotional dysfunction (Bremer et al., 2016).

Given the increasing prevalence of ASD and the lifelong nature of this condition, as well as the trend of reduced physical exercise in the transition to adulthood (McCoy et al., 2016; Ratcliff et al., 2018; Sung et al., 2023), identifying the most beneficial type of physical exercise program for individuals with ASD, offering evidence-based guidelines in the prescription of exercise aimed at improving the health and well-being of the population with ASD, is crucial. In addition, the current literature reveals a significant gap in specific guidelines on exercise programs aimed at young people and adults with ASD, especially in the 18–30-year-old age group, with only three systematic reviews including participants in this age group (Orr et al., 2020; Shahane et al., 2023; Sung et al., 2023).

The aim of this systematic review is to answer the following two questions: (i) What type of exercise program is most beneficial for individuals with ASD: cardiorespiratory, muscle strength or combined? and (ii) What are the most common and effective guidelines for prescribing exercise programs for individuals with ASD?

## Methods

### Research Strategies

This systematic review was carried out in 2024 using five databases: Education Resources Information Center (ERIC); Web of Science; PsycINFO; PubMed; and Scopus. These databases use descriptors indexed in the Medical Subject Headings: “(Autism Spectrum Disorder” OR “Autism Spectrum” OR “Autism)” AND “(Resistance Training” OR “Strength Training” OR “Neuromuscular Training” OR “Resistance Exercise” OR “Strength Exercise” OR “Neuromuscular Exercise)”.

### Data Selection Criteria

#### Inclusion Criteria

The studies were selected on the basis of the following criteria: (i) research published between 2003 and 2024; (ii) pilot and experimental studies (randomized clinical trials or single subject designs), published in peer-reviewed journals; (iii) studies with a sample of children, young people and/or adults diagnosed with ASD (autistic disorder; Asperger's syndrome and pervasive developmental disorder without other specification), in different le vels; (iv) no restrictions on ethnicity, gender, age group, sample number or duration of the physical exercise program; (v) studies that used cardiorespiratory fitness and/or muscular strength as the dependent variable; (vi) studies that have assessed cardiorespiratory fitness and/or muscle strength; and (vii) studies that have implemented a group physical exercise program as an independent variable.

#### Exclusion Criteria

The studies were excluded on the basis of the following criteria: (i) articles published before 2003; (ii) articles not published in English, Spanish or Portuguese; (iii) systematic review, meta-analysis and/or thesis/dissertation articles; (iv) studies that covered other types of disability without ASD; (v) studies that did not use cardiorespiratory fitness and/or muscle strength as a dependent variable; (vi) studies that did not assess cardiorespiratory fitness and/or muscle strength; and (vii) studies that did not describe the intervention protocol, namely, the physical exercise program.

#### Data Extraction

The articles were imported into the software (EndNote v.20.2.1, London, UK), and duplicates were removed. The selection of the studies was performed by two independent reviewers in phases: (i) reading the titles and abstracts; (ii) complete reading of the articles; (iii) classifying the articles, categorizing them as included, excluded and doubtful; and (iv) checking the quality of the information in each study as a final eligibility criterion.

### Methodological Design

This systematic review was conducted in accordance with the Preferred Reporting Items for Systematic Reviews and Meta-Analyses (Page et al., 2021; Rethlefsen et al., 2021). These guidelines describe the three phases (identification, screening and final selection) to be followed to carry out the research. Moreover, the PICOS approach was used, which directed the refinement of the study, making it a more effective process (Panic et al., 2013). The main qualitative and quantitative data from the selected articles were subsequently extracted and organized in a specific table according to the PRISMA method. The items obtained include author, year, parents; objectives; sample (level of functionality, number of participants, gender and age); type of program; intervention protocol (exercise, duration, frequency, volume and intensity); and evaluation tools/techniques and results. Notably, the entire process described was carried out by two reviewers.

### Quality of Information

After the first selection, the studies were evaluated using the dichotomous PEDro scale (Moseley et al., 2002). This scale has a total of 11 evaluative items, with the exception of item no. 1, which gives the study 1 point for each item met, totaling 10 points. Items 2 to 9 analyze the study's internal validity, whereas items 10 and 11 evaluate its statistical characteristics so that the results can be interpreted. The risk of bias (ROB) was assessed using the PEDro scale (Moseley et al., 2002). Studies were classified as having “low (L)” ROB (score ≥ 6) or “high (H)” ROB (score < 6) on the basis of the total PEDro score.

## Results

### Study Selection

**Fig. 1:**
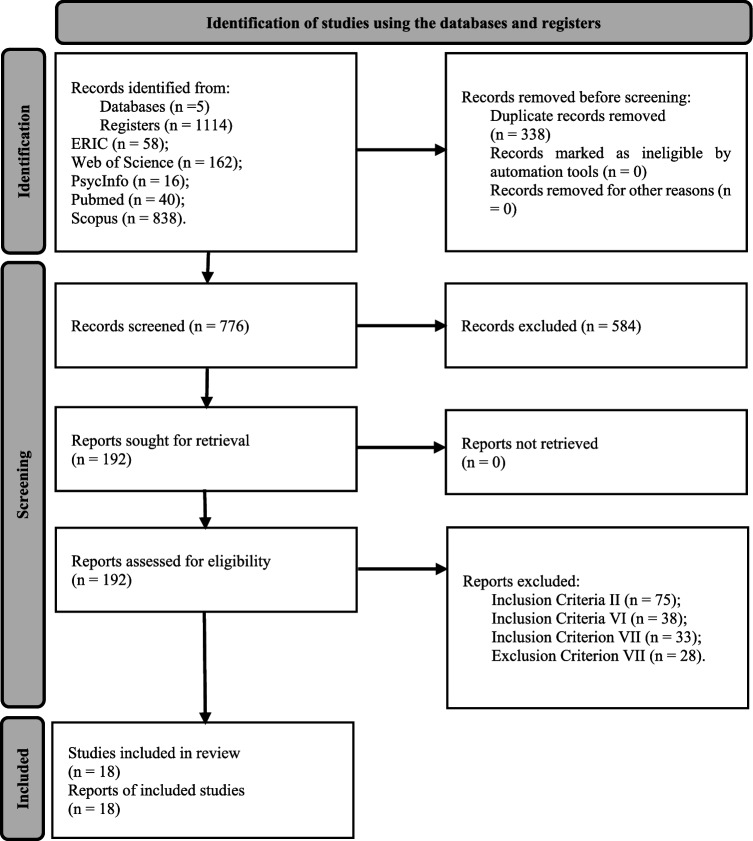
PRISMA 2020 flowchart-research strategy and study selection process

A total of 1114 studies were identified by searching 5 databases. In the first phase, which included reading the titles and abstracts, 192 potentially relevant studies were identified. Considering the previously defined eligibility criteria and after the articles were read in full, 18 studies that met the eligibility criteria were identified and included in a full analysis. Flowchart 1 presents information on the three phases of the systematic search, which is based on the PRISMA guidelines (Page et al., 2021; Rethlefsen et al., 2021) (see Fig. 1).

### Study Characteristics

As shown in Table 1, the PEDro scale was applied to the 18 eligible articles to assess the quality of their methodology. In this regard, we identified (n = 11) studies classified as having a low ROB, which corresponds to scores between 6 and 10, and (n = 7) studies classified as having a high ROB, with scores between 1 and 5.

**Table 1:** Quality assessment of the articles using the PEDro Scale, resulting in a total score

Citation	PEDro scale											Score (0–10)	ROB*
	Eligibility criteria specified	Random subject allocation	Concealed allocation	Baseline similarity of groups	Blinding: subjects	Blinding: therapists	Blinding: assessors	Measures of key outcomes	Intent to treat	Between group analyses	Point estimates and variability measures		
Lochbaum and Crews (2003)	1	0	1	0	1	1	1	1	0	0	0	5	H
Fragala Pinkham et al. (2005)	1	0	0	0	0	0	0	1	0	0	1	2	H
Todd and Reid et al. (2006)	0	1	1	1	0	0	0	1	0	0	1	5	H
Pitetti et al. (2007)	1	0	0	1	0	0	0	1	0	1	1	4	H
Magnusson et al. (2012)	1	0	0	1	1	0	1	1	0	1	1	6	L
Bricout et al. (2018)	1	1	1	1	1	0	1	1	0	1	1	8	L
Shavikloo and Norasteh (2018)	1	0	1	1	1	0	1	1	0	1	1	7	L
Shields et al. (2018)	1	0	0	0	0	0	0	1	0	1	0	2	H
Spratt et al. (2018)	1	1	1	1	1	0	1	1	1	0	0	7	L
Toscano et al. (2018)	1	1	1	1	1	0	1	1	0	1	1	8	L
Jimeno (2019)	1	0	0	1	0	0	0	1	0	0	1	3	H
Todd et al. (2019)	0	1	1	0	1	0	1	1	0	0	0	5	H
Arslan et al. (2020)	1	1	1	0	0	0	0	1	1	1	1	6	L
Cha et al. (2020)	1	0	0	1	1	0	1	1	1	1	1	7	L
Kozlowski et al. (2021)	1	1	1	1	1	0	1	1	0	1	1	8	L
Yu and Jee (2020)	1	0	0	1	0	1	1	1	0	1	1	6	L
Bittner et al. (2021)	1	1	1	1	1	0	1	1	0	0	1	7	L
Savage et al. (2022)	1	1	0	1	0	0	0	1	1	1	1	6	L

^*^low risk = score ⩾6 (l) and high risk = score < 6 (h). The scores of these items are added together to provide a total pedro score (maximum score = 10); item 1 is not included in the calculation of the total score

#### Origin

With respect to origin, Australia (n = 2), Brazil (n = 1), California (n = 1), Canada (n = 1), Korea (n = 2), the United States of America (n = 7), France (n = 1), Iran (n = 1), New Zealand (n = 1) and Turkey (n = 1) were the places where the research included in the review took place, with the United States of America being the country with the most publications on the subject.

#### Participants

The participants included in the studies were selected on the basis of the following criteria: (i) a closed diagnosis of autism spectrum disorder (autistic disorder; Asperger's syndrome and pervasive developmental disorder without other specification), proven in an institutional clinical report, in accordance with the standards established by the Diagnostic and Statistical Manual of Mental Disorders—5th edition; (ii) absence of other syndromes and motor incapacity conditions associated with ASD; (iii) good health condition, compatible with regular physical exercise, certified by a doctor; (iv) ability to undergo psychological assessment tests; and (v) free use of medication.

The total sample consisted of 445 participants, of whom 187 were male and 18 were female. In 9 studies, specific data on the sex split were not available. The training group included 74 participants, whereas the control group included 94 participants. The literature reviewed revealed wide variability in the ages of the participants, -57 years. Although the main focus was on young people and adults aged 18–30, only 4 studies included this age group. More than half of the studies included children, and in 2 of them, the ages of the participants were not specified. The level of functionality of the participants was mentioned in only 2 studies, both of which applied exercise programs for severe levels of functionality in individuals with ASD, whereas the remaining 16 studies did not provide this information. The recruitment of participants took place predominantly in private social solidarity institutions (PSSIs) in therapeutic and controlled environments, as reported in 14 studies. The remaining 4 studies described interventions involving physical exercise in a school environment.

#### Type of Programme

On the basis of the studies reviewed, 13 of the 18 exercise programs aimed at individuals with ASD applied combined programs, which were the most prevalent. These programs integrated exercises aimed at improving both cardiorespiratory fitness and muscle strength. Progression on the aerobic side included continuous walking, moving on to continuous running and evolving to variable speed running, whereas the muscular strength side followed initial work on muscular endurance, progressing to endurance strength. In addition, 4 programs exclusively implemented cardiorespiratory fitness programs, and only 1 program focused solely on developing muscular strength.

#### Intervention Protocols

The exercise programs that assessed cardiorespiratory fitness or muscle strength separately had fairly disparate durations, between 6 weeks and 6 months (n = 3), and 2 studies did not report this information. The frequency varied between 2 and 3 times a week (n = 5), with the most common weekly frequency being 3 times (n = 2), and the duration that varied between 8 and 30 min (n = 5) was determined using the circuit dynamics (n = 2), with the following distributions: warm-up (5 min); main part (20 min) and return to calm (5 min). The exercise program that only investigated strength training methodologies asked for the main muscle groups in each session. With 2 sets, the number of repetitions varied between 7 and 10 for dynamic exercises and 10–30 repetitions for static exercises (n = 1) per set. The initial speed on the treadmill was 2.4 mph to 3.5 mph, with a progression of 0.1 mph to 0.3 mph every 2–3 weeks and a maximum speed between 3.7 and 4.1 mph (n = 1). The combined training programs lasted from 14 days to 48 weeks, with 12 weeks being the most common (n = 5). The programs were carried out between 1 and 3 times a week, with twice a week (n = 7) being the most common, followed by 3 times a week (n = 2). The training sessions ranged from 20 to 60 min, with the majority of professionals prescribing 60 min per session (n = 4), followed by 45 min (n = 2). Aerobic exercise was carried out at a moderate to vigorous intensity of 50–60%, progressing to 75–80% of HRM. Muscle strength was started at 60% of the maximum weight, performing 1 set of 6 to 15 repetitions, with an increase of approximately 5% per week, up to 80% to 85% of the maximum weight, and performing 3 sets of 6 to 8 repetitions (n = 2). The target population had deficits in physical qualities, and the most common types of interventions were walking (n = 12), treadmill walking (n = 2), cycling or cycle ergometer training (n = 3), going up and down steps (n = 1), obstacle courses and handling objects (n = 2) and motor coordination training (n = 1). With respect to muscle strength training, there was an increase in the load throughout the programs, which used resistance bands, shin guards or free weights in exercises such as plyometrics, step-ups, press-ups, curl-ups (n = 1), climbing and holding the bar (n = 1). There was a pattern in the movement and, in turn, a pattern in the main muscle needed, the most common being leg flexion (hamstring), leg extension (quadriceps), abs (abdominal muscles), vertical pull (great dorsal), arm flexion (biceps), arm extension (triceps) and the shoulder lift (deltoid) (n = 1).

#### Evaluation Instruments/Techniques

In the studies analyzed, the diagnosis of ASD was made using the Autism Diagnostic Observation Scale (ADOS) (n = 1), the Gilliam Autism Rating Scale 2nd or 3rd edition (GARS) (n = 1) or the Childhood Autism Rating Scale (CARS) (n = 1). The Child Health Questionnaire-Parent Form (CHQ-PF50) (n = 1), the Patient Health Questionnaire-9 (PHQ-9) (n = 1) and the Pediatric Quality of Life Inventory (PedsQL) (n = 1) were used to measure health-related quality of life.

The anthropometric assessment was cross-sectional in most of the studies, with the variables weight, height, waist circumference, waist-to-height ratio and body mass index being collected (n = 3). Some studies, with the aim of methodological enrichment, opted to include the collection of biological markers from blood samples (n = 2) and glucose (n = 1) and assess fat-free mass and fat mass through electrical bioimpedance (n = 2). The anthropometric measurements were taken separately in a private room following standardized procedures. A preassessment was identified in the various studies analyzed, in which they used the Brockport Test Battery (n = 1), the Bruininks–Oseretsky Motor Proficiency Test (n = 1), the Gross Motor Function Classification System (GMF-II) (n = 1) or the Test of Gross Motor Development (TGMD) (n = 1).

A transversal factor in cardiorespiratory exercise programs is the direct determination of VO^2^ Max through incremental, continuous and submaximal protocols, such as the modified Bruce protocol (n = 1), or through functional tests, such as the shuttle-run test (n = 2). Neuromuscular capacity was mostly assessed using the handgrip strength test, as well as the modified curl up and push up test (n = 7). HR was monitored manually (n = 2) or using the Polar RS800CX frequency meters (n = 1). Thus, in the studies that monitored HR, exercise intensity was predicted indirectly usging the formula HRM = 207 – 0.7 × age (Gellish et al., 2007). In addition, two studies used behavioral indicators (red face, rapid breathing or stop jumping for 1 min) to assess effort levels during exercise.

#### Results

The results regarding body composition revealed that the training group (TG) presented a significant reduction in body mass index (BMI), ranging from 2.8 to 17.2%, compared with baseline. In contrast, the control group (CG) showed smaller reductions in BMI, ranging from 3.0 to 9.6% (Pitetti et al., 2007). The results related to cardiorespiratory fitness revealed that the TG participants progressively increased the distance covered, ranging from 0.83 to 1.26 km over the course of the sessions, despite the reduction in verbal reinforcement (Todd & Reid, 2006). However, the VO^2^ Max, duration and maximum speed values were significantly lower in the TG than in the CG (p < 0.05), which indicated a lower cardiorespiratory fitness in individuals with ASD than in their peers with typical development (Bricout et al., 2018). Despite this, the TG demonstrated important advances, such as an increase in daily steps, significant weight loss compared with the CG, and a 33% increase in aerobic fitness (Lochbaum & Crews, 2003). With respect to muscle strength, significant increases in work output were observed in three tests: sit ups, air squats and long jump (Kozlowski et al., 2021). A comparison of the pre- and posttest results revealed that when compared with the CG, the TG (p < 0.05) showed 30% greater development in terms of balance, long jump, reaction time and handgrip strength (Arslan et al., 2020). Strength gains resulted in improvements in anterior, post-medial and post-lateral movements (p ≤ 0.05), indicating that the intervention had a positive effect on postural stability (Shavikloo & Norasteh, 2018) with strength increases of between 19 and 28% (Lochbaum & Crews, 2003). The results confirm the effectiveness of exercise programs in improving body composition, cardiorespiratory fitness and muscle strength in individuals with ASD. In addition to the physical benefits, the interventions also promoted significant improvements in mental health and quality of life, with a reduction in depression rates (Spratt et al., 2018) and an increase in the psychosocial health score, that ranged from 9.8 to 20.7 (Toscano et al., 2018). Tables 2 and 3 show the author/year/parents, objectives, participants, type of program, intervention protocol, evaluation instruments/techniques and main results of the included studies.

**Table 2:** Summary of articles reviewed on the effects of exercise programs on cardiorespiratory fitness or muscle strength in individuals with ASD

Author, year and country	Aims	Participants	Type of program	Intervention protocol	Evaluation instruments/techniques	Results
Todd, 2006, Canada	To investigate the effects of a jogging intervention on the physical fitness of young people with ASD	N = 03; (M) 15–20 years	Cardiorespiratory fitness	Exercise: walking/running; Duration: 6 months; Frequency: 2 times a week; Volume: 30' per session	The evaluation was carried out through self-monitoring, with monitors and participants recording the number of circuits completed during the program	Three participants increased their distance covered over the course of the sessions (.83 km–1.14 km–1.26 km), while verbal reinforcement was reduced
Pitetti, 2007, USA	To evaluate the effectiveness of a nine-week treadmill walking program for individuals with ASD	Severe ASD TG = 10; (4 F; 6 M) CG = 32; NI	Cardiorespiratory Fitness	TG Exercise: Walking on the treadmill; Frequency: 5 times a week; Volume: 8' to 20'; Intensity: An initial treadmill speed of 2.4 mph to 3.5 mph, with a progression of 0.1 mph to 0.3 mph every 2–3 weeks and a peak speed ranging from 3.7 mph to 4.1 mph CG Exercise: basketball; skating; cycling and jumping rope; Frequency: 3 times a week; Volume: 30 min per session	I. BMI was calculated using the standard formula: Weight/Height (kg/m^2^); II. Caloric expenditure on the conveyor belt was calculated as follows: VO^2^ = .1 (Speed of TR) + 1.8 (Fractional Degree of TR) + 3.5; III. Then, converted to METS by dividing the VO^2^ value by 3.5 ml/kg-^1^/min-^1^ and the calorie expenditure per minute, calculated using the formula: Kcal min-^1^ = (METS—3.5—Body Weight)/200	The comparison between baseline and week nine showed the following; I. In the TG, the percentage change in BMI compared to baseline indicated that there was at least a 2.8% reduction up to 17.2%; II. In the CG, only three participants reduced their BMI, corresponding to a percentage variation of 3.0% to 9.6%
Bricout, 2018, France	To compare the physical fitness of young people with ASD in relation to young people with typical development, through VO^2^ Max	TG = 20; M (10.7 ± 1.2) CG = 20; M (10.0 ± 1.6)	Cardiorespiratory Fitness	The intervention involved the use of a SenseWear® Pro-3 Accelerometer for a period of 7 days during a typical school week; The instrument was used 24 h a day for all activities except bathing and swimming	I. The diagnosis of ASD was made using the ADOS Scale; II. VO^2^ Max was measured using an incremental, continuous and maximal protocol on a treadmill; III. Blood lactate samples were taken	I. VO^2^ Max, duration and maximum speed values were significantly lower in the TG compared to the CG (*p* < .05); II. The young people with ASD showed lower cardiorespiratory fitness than the sample with typical development, despite similar levels of physical activity; III. The results suggest that the difference may stem from motor discrepancies
Shavikloo, 2018, Iran	To evaluate the effect of neuromuscular training on postural control in children with ASD	TG = 12; (M) CG = 12; (M) 6–10 years	Muscle Strength	TG Exercise: integrative neuromuscular training; Duration: 6 weeks; Frequency: 3 times a week; Structure: Warm-up (5 min), followed by five primary exercises, which focused on increasing the strength of the lower and upper limbs; Number of sets: 2; Number of repetitions: 7 to 10 repetitions in the dynamic exercises and 10 s to 30 s in the static exercises CG Exercise: Normal exercise routine; Duration: 6 weeks; Frequency: 3 times a week	I. The diagnosis of autism spectrum disorder was made using the Gilliam Autism Rating Scale; II. The dynamic and static balance assessment was carried out in a laboratory environment using a balance platform	I. The results indicated a significant difference in SEBT and BESS during the six weeks for the TG (*p* ≤ .05); II. The results indicated significant differences in three movements (anterior, post-medial and post-lateral) in the TG (*p* ≤ .05); III. The neuromuscular training program has a positive effect on postural control ability in young people with ASD
Savage, 2022, USA	To evaluate the effectiveness and viability of the “Step It Up” program in a group of adults with ASD	TG = 18; NI CG = 16; NI CCG = 17; NI 18–57 years	Cardiorespiratory Fitness	Exercise: Walking monitored by Fitbit; Frequency: Daily; Intensity: Moderate	I. Severity of ASD—CARS; II. Weekly Step Count—Fitbit; III. Fitbit Evaluation by Trainers: usage rating profile; IV. Revised Intervention Evaluation—Assessing participants opinions on the feasibility and acceptability of the intervention	TG Results—Higher step count and significant weight loss compared to CG; Feasibility and Acceptability—High level of feasibility and acceptability of the home program

*min* minutes, *%* percentage, *ADOS* autism diagnostic observation schedule, *ASD* autism spectrum disorder, *BESS* static balance platform, *BMI* body mass index, *BMR* basal metabolic rate, *CARS* childhood autism rating scale, *CCG* coaching group, *CG* control group, F total female sample, *GMF* gross motor function classification system, *kcal* kilocalorie, *km* kilometers, *m* meters, *M* total male sample, *met* metabolic equivalent of the task, *mph* miles per hour, *n* number of participants, *NI* gender of the sample not identified, *s* seconds, *SEBT* dynamic balance platform, *TG* training group, *TR* treadmill, *USA* United States of America, *VO*^*2*^ oxygen consumption, *VO*^*2*^* max* maximum oxygen consumption

**Table 3:** Summary of the articles reviewed on the effects of combined training programs on individuals with ASD

Author, year and country	Aims	Participants	Type of program	Intervention protocol	Evaluation instruments/techniques	Results
Lochbaum, 2003, USA	To investigate the effects of a physical exercise program according to standard guidelines in mild ASD	N = 05; M 16–21 years	Combined	Exercise: aerobic (cycle ergometer) and muscular strength (bodyweight and free weights); Duration: 6 weeks; Frequency: 3 sessions per week; Volume: 20 min per session; Intensity: Aerobic exercise was performed at a moderate intensity—65% to 70% of HRM. Muscle strength was started at 60% of maximum weight, performing 3 sets of 12 repetitions. The intensity was increased by approximately 5% per week, up to 80% to 85% of the maximum weight, performing 3 sets of 6 to 8 repetitions	I. HR was monitored with a cardio frequency meter; II. Changes in muscle strength were determined before and after training and measured using 1 MR maximal lifts	I. Aerobic fitness increased by 33%, 50% and 33% for the 3 participants in the sample; II. Muscle strength increased by 19% and 28% for the 2 participants, respectively; III. In this study, aerobic training improved the physical fitness of young people with ASD significantly more than strength training
Fragala Pinkham, 2005, USA	To describe a physical fitness program for young people with autism spectrum disorder, providing information on the feasibility of the intervention	N = 09; NI 5–9 years	Combined	Exercise: The program involved a combination of aerobic and muscle strength training, aimed at obstacle courses, object manipulation and body exercises; Duration: 26 sessions, 14 in a group and 12 at home; Frequency: 2 times a week; Intensity: FC between 50%-60% of FCM and progressing to 75%-80%; Number of sets: 1 set; Number of repetitions: 6 to 15 repetitions	Cardiovascular fitness—shuttle-run test; muscular strength—isometric dynamometry; and flexibility—sit and reach test	I. 6 out of 9 young people improved their cardiovascular results; 7 out of 9 improved their lower body muscle strength; II. More positive improvements were observed after the group exercise program than after the home exercise program
Magnusson, 2012, New Zealand	To investigate whether an individualized high-intensity program will have a positive effect on the physical fitness and behaviors of young people with autism spectrum disorder	N = 06; NI 9–15 years	Combined	Exercise: step-ups, press-ups and curl-ups; Duration: 8–12 weeks; Frequency: 2 times a week; Intensity: high intensity combined training	Cardiovascular fitness—modified Bruce protocol; muscular strength – 1 MR test; flexibility—sit-and-reach test; balance—modified Romberg test	There were significant improvements in cardiovascular fitness, abdominal strength and the frequency of problem behaviors
Shields, 2018, Australia	To evaluate the feasibility of an exercise program for young people with ASD	N = 19; (9F;10 M) X = 18 years	Combined	Exercise: The main exercise sequences were aimed at muscle toning, cardiovascular improvement and postural control; Duration: 12 weeks; Frequency: twice a week; Volume: 45 to 60,min per session	Five areas of feasibility were assessed: demand; implementation; practicability; limited effectiveness testing and acceptability	I. Training fidelity showed significantly higher exercise intensity in Aerobic and Strength exercises; II. The limited efficacy tests showed an increase in UL strength (4 kg, 1–7) and LL strength (43 kg, 24–62), walking endurance (80 m, 24–137), as well as improvements in three dimensions of quality of life—autonomy, physical and psychological well-being
Spratt, 2018, USA	Developing the “Piece It Together” program, fostering socialization and well-being goals in young people with ASD	N = 11 ASD; 1 ID (5F; 7 M) 15–27 years	Combined	Exercise: Yoga, meditation, deep breathing and stretching; Duration: 12 weeks; Frequency: twice a week; Structure: Classes included exercise (45–60 min), stress reduction (15–30 min) and nutritional education (15–30 min)	The PHQ-9 was administered to each participant at the beginning and end of the intervention	I. Significant reduction in depression scores (PHQ-9), from mild to minimal (*p* = .000); II. Additional results included a statistically significant increase in skeletal muscle mass
Toscano, 2018, Brazil	Effects of a 48-week exercise-based intervention on weight, metabolic profile, symptomatological characteristics and health-related quality of life in children with ASD	N = 46; NI 8.2 ± 1.7 years	Combined	Exercise: climbing; handstand; basket throwing; elastic band work; step walking; step box with target and sequenced walking; Duration: 48 weeks, 92 sessions; Frequency: 2 times a week; Volume: 40 min per session	I. Anthropometric measurements: height, body mass, BMI, waist circumference and waist-to-height ratio; II. Biological markers were assessed using blood samples, using the Friedewald formula; III. Glucose was assessed using the enzymatic method (Glucose Oxidase – Laboratory test); IV. The CARS-BR Childhood Autism Rating Scale was used, in the Brazilian Portuguese version; V. The children's legal guardians filled in the Portuguese version of the CHQ-PF50	I. There was an increase in HDL and a decrease in LDL; II. For both the autistic traits scale (-12.2 to -4.0) and the motor profile scale (-3.3 to -1.5), reductions in symptoms were observed after the 48-week intervention; III. As for the quality of life perceived by the parents of children with ASD, the intervention group showed an increase in both the physical health score (7.7 to 18.9) and the psychosocial health score (9.8 to 20.7)
Jimeno, 2019, Australia	To investigate the effects of a neuromuscular training program on young people with ASD	N = 04; NI 9–16 years	Combined	Exercise: Involved aerobic exercise, muscle strength, plyometrics, balance, motor coordination and the development of social skills; Duration: 16 weeks, divided into two 8-week blocks	I. Data were collected from the reports of participants and legal guardians using PedsQL; II. Six months after the end of the program, PedsQL was applied once again in order to understand what changes had occurred in the long term	The results showed an increase in motor skills and quality of life, both functionally and psychosocially
Todd, 2019, USA	Effects of a physical activity intervention on health-related issues, fitness levels and adherence rates	N = 16; NI	Combined	Exercise: The Into Fitness Together (IFiT) program was applied to university students with ASD, offering individualized physical activities—aerobics and muscle strength; Duration: 10 weeks; Frequency: twice a week; Volume: 2.5 h per session	I. Anthropometric and fitness measurements before and after the IFiT program; II. Mentors recorded details of the sessions, including type of activity, location and repetitions	I. Students with ASD and their peers improved cardiorespiratory fitness, flexibility and upper muscular endurance with the IFiT program; II. Initially, most of the participants were overweight, after IFiT, there were significant improvements; III. High adherence (89.1%), a factor indicating consistency over the course of the sessions
Arslan, 2020, Turkey	To determine the effects of a circuit exercise program on physical fitness parameters in children with autism spectrum disorder	TG = 14; (M) 10.07 ± 0.25 CG = 14; (M) 10.07 ± 0.30	Combined	The children were divided into two groups—exercise (TG) and control (CG); Duration: 12 weeks; Frequency: 3 sessions per week; Volume: 60 min per session	The sample was assessed according to the parameters of the Bruininks–Oseretsky test and gross motor proficiency, which included speed, agility, balance and bilateral coordination	Comparing the pre- and posttest results of the TG and CG, the former showed 30% greater development in terms of balance, long jump, reaction time and handgrip strength (*p* < .05)
Cha, 2020, Korea	Analyze the differences between gross motor function and physical fitness	ASD N = 15 (M) ID N = 14 (M) 19–28 years	Combined	Exercise: The program began with a conditioning warm-up (5 min); then 1 type of session (40–45 min) was chosen: routine games (basic locomotion skills; catching; tug-of-war game); floorball games (receiving and passing the ball exercises; passing practice; short mini-game); basketball (dribbling; passing; throwing the ball) or inline skating (basic walking, pushing and turning skills). the program ended with 10 min of stretching; Duration: 1 year; Frequency: 2 days a week	I. Electrical bioimpedance: bmi, muscle mass, fat mass, lean mass, waist to hip ratio and basal metabolic rate; II. Height; III. Flexibility—sit and reach test IV. Grip Strength—digital hand dynamometer; V. Cardiorespiratory Endurance—15 m shuttle-run and personalized abs test	I. There were no statistically significant differences between groups in terms of gross motor function (locomotion; ability to control objects) and physical fitness (body composition; flexibility; muscle strength and cardiorespiratory endurance); II. The study indicates that physical exercise programs with a certain structure can have similar effects, even if they contain different areas of disability, with similar comorbidities;
Kozlowski., 2020, USA	To investigate the feasibility of applying a high-intensity program to young people with autism spectrum disorders	N = 58; NI 7–12 years	Combined	Exercise: The program covered the following skills: endurance; strength; flexibility; balance; motor coordination; speed, agility; power and precision; Duration: 5 weeks, corresponding to 19 sessions; Frequency: 1 time per week; Intensity: moderate to vigorous; Structure: The structure consisted of a 5 min instruction period, a 7–10 min warm-up, a 15–20 min main part and a 5 min return to calm	I. Results were assessed through biometric measurements, physical activity levels (Actigraph GT3X accelerometer), and exercise performance; II. Physical fitness was assessed at baseline and postprogram through a set of physical tests chosen for this purpose (pacer, sit and reach, push-ups, sit-ups, air squats and long jump)	I. Significant increases in work output and activity levels (.83 and 1.05, respectively) were found in three tests: sit ups, air squats and long jump (.29-.37); II. The results indicated high levels of fidelity (93.7%) and satisfaction among the young people, and there was no attrition or injury, supporting the viability of the protocol
Yu, 2020, Korea	To investigate the effects of an exercise program on the physical fitness of adults with ASD	Mild ASD (N = 17; M) Severe ASD (N = 18; M) 22.83 ± 2.72	Combined	Exercises: Walking and motor coordination; Duration: 12 weeks; Frequency: 2 times a week; Structure: This program was divided into a 5 min warm-up, followed by 45 min of training and, finally, 10 min of stretching	I. The GMF-II scale was used to measure delays in movement patterns compared to their peers; II. Analysis of body composition using electrical bioimpedance; III. Assessment of cardiorespiratory capacity—shuttle run test (15 m); flexibility—sit and reach test; grip strength—hand dynamometer; muscular power—long jump	In the mild ASD group, locomotion function increased by 19.81%, while in the severe ASD group it decreased by 4.78%. Object control improved by 29.96% in the mild group, but decreased by 15.2% in the severe group
Bittner, 2021, California	Quantify the intensity of exercise resulting from participation in “Camp Nugget”	N = 18; NI 5–12 years	Combined	Exercise: The younger groups (5–9 years old) took part in skydiving, hula hooping or cycling. The older groups (10–12 years old) took part in leadership sports (soccer, volleyball and basketball); Duration: 14 days; Structure: The physical activity routine was categorized by the following activities: warm-up; individual time; group station and aquatic classes	I. The height and weight of the participants were quantified using a digital scale and a portable stadiometer; II. A preassessment of physical activity was carried out using the Brockport Test Battery; III. Participants were acclimatized to the Actiheart monitor over a period of three days. This device uses a piezoelectric accelerometer with a synchronized system to measure HR and estimate EE	I. The overall program completion rate was 77.32%; II. The average HR during the 14 sessions was 62.2%—63.5%; III. The average EE was 3.4 to 3.6 MET, over all of the sessions

*min* minutes, *%* percentage, *ACS-ASD* anxiety scale for children with autism spectrum disorder, *ASD* autism spectrum disorder, *BMI* body mass index, *BPFT* Brockport physical fitness test, *CARS* childhood autism rating scale, *CARS-BR* childhood autism rating scale Brazil, *CG* control group, *CHQ-PF50* child health questionnaire, *EE* energy expended, *F* female, *GT* training group, *HDL* high-density lipoproteins, *HR* heart rate, *ID* intellectual disability, *kg* kilograms, *LDL* low-density lipoproteins, *ll* lower limbs, *m* male, *m* meters, *M* total male sample, *MET* metabolic equivalent of the task, *MHR* maximum heart rate, *MR* repetition maximum, *n* number of participants, *NI* gender of the sample not identified, *PEDSQL* pediatric quality of life inventory, *PHQ-9* patient health questionnaire, *s* seconds, *TGMD* test of gross motor development, *UL* upper limbs, *USA* United States of America, *x* average in years

## Discussion

The aim of this systematic review is two folded. First, to analyze which type of exercise program is most beneficial for individuals with ASD—cardiorespiratory, muscle strength or combined programs and secondly, to identify the characteristics of the prescription programs analyzed, namely, duration, weekly frequency, intensity and the most appropriate assessment methods. The following subsections cover all the main points of the exercise programs analyzed.

### Type of Program

As mentioned in the Results section, 13 of the 18 exercise programs aimed at individuals with ASD used combined programs, making them the most prevalent. In this sense, the results indicated improvements in the participants' lipid profile with an increase in high-density lipoprotein (HDL) levels and a reduction in low-density lipoprotein (LDL) levels (Toscano et al., 2018). With respect to cardiorespiratory fitness, there was an increase of up to 50%, whereas muscle strength increased by up to 28% (Lochbaum & Crews, 2003). With respect to motor skills, participants with mild ASD showed a significant improvement in locomotor function with an increase of 19.81% (Yu & Jee, 2020). In the study by Todd et al. (2019), a 30% greater development in motor skills was observed between the pre- and postintervention tests. In terms of mental health, there was a significant reduction in depression scores, as indicated by the PHQ-9, as well as a decrease in the frequency of problem behaviors (Spratt et al., 2018). The physical and psychosocial health scores increased from 7.7 to 18.9 and from 9.8 to 20.7, respectively (Toscano et al., 2018). Thus, the results confirm the effectiveness of combined exercise programs in improving body composition, cardiorespiratory fitness, muscle strength, motor capacity, and mental and psychosocial health in individuals with ASD (Arslan et al., 2020; Bittner et al., 2021; Cha et al., 2020; Fragala Pinkham et al., 2005; Jimeno, 2019; Kozlowski et al., 2021; Lochbaum & Crews, 2003; Magnusson et al., 2012; Shields et al., 2018; Spratt et al., 2018; Todd et al., 2019; Toscano et al., 2018; Yu & Jee, 2020).

### Duration

As previously mentioned in the Results section, the duration of the combined programs ranged from 14 days to 48 weeks, with 12 weeks being the most common duration (Arslan et al., 2020; Magnusson et al., 2012; Shields et al., 2018; Spratt et al., 2018; Yu & Jee, 2020). Short-term intervention programs may be a limitation in some studies (Li et al., 2024). Although the 13 studies reviewed presented several positive results (Table 3), more studies with extended durations are needed for a better understanding of the long-term benefits, as well as the ideal type of combined periodization to be applied to the ASD population. Recent studies indicate that the duration of training programs can be a crucial factor in the success of interventions involving physical exercise in individuals with ASD. Short duration programs may not provide enough time for physiological and psychological adaptations to occur, which may explain why some individuals do not show significant improvements. Therefore, it is essential to consider that interventions of longer duration may favor a more significant accumulation of physical benefits, such as increased endurance and muscle strength, in addition to improvements in mental health, promoting more consistent and sustainable results over time (Toscano et al., 2022).

### Frequency

The weekly frequency of aerobic exercise followed the recommendations of the American College of Sports Medicine (ACSM) (2022), with 2 to 3 sessions per week for aerobic exercise and 1 to 2 sessions per week for muscle strength work (Fragala Pinkham et al., 2005; Magnusson et al., 2012; Shields et al., 2018; Spratt et al., 2018; Todd et al., 2019; Toscano et al., 2018; Yu & Jee, 2020). This number of weekly sessions favors adaptations that lead to catabolism followed by protein anabolism, allowing lean mass to be maintained or increased (Toscano et al., 2018). In addition, with the exception of the study by Bittner et al. (2021), all of the other studies combined avoided sessions on consecutive days, and the study by Jimeno (2019) did not specify the frequency of sessions.

### Session Volume

The volume of the training sessions varied between 45 and 60 min for aerobic exercises and 20 min for strength exercises, using a dynamic circuit, with the following distribution: warm-up, main part and return to calm in line with the ACSM guidelines (2022). However, some studies do not mention the duration of the sessions (Bittner et al., 2021; Cha et al., 2020; Fragala Pinkham et al., 2005; Jimeno, 2019; Magnusson et al., 2012) which is a limitation, leaving some doubts about the recovery period that was applied, as well as the replicability of the studies.

### Sets

The prescription of 1 to 3 sets was more frequent (Fragala Pinkham et al., 2005; Lochbaum & Crews, 2003) and in accordance with the ACSM (2022). Some authors stated that in untrained individuals both single sets and multiple sets produced similar increases in upper and lower limb muscle strength, i.e., in the initial stages, strength training, regardless of the number of sets, seems to be effective in improving muscle results (Fragala Pinkham et al., 2005; Lochbaum & Crews, 2003).

### Repetitions

The number of repetitions per set varied between 6 and 12 for moderate-intensity exercises and between 6 and 8 for vigorous-intensity exercises (Lochbaum & Crews, 2003; Fragala Pinkham et al., 2005), being influenced by the prescription of one repetition maximum (1 RM) methods. This figure is in line with the recommendations of the ACSM (2022). However, more than half of the studies did not mention the number of repetitions (Arslan et al., 2020; Bittner et al., 2021; Cha et al., 2020; Jimeno, 2019; Kozlowski et al., 2021; Magnusson et al., 2012; Shields et al., 2018; Spratt et al., 2018; Todd et al., 2019; Toscano et al., 2018; Yu & Jee, 2020), which raises doubts about the recovery period applied and the replicability of the studies.

### Intensity

The combined programs had different training intensities, adjusted according to the objectives. Aerobic exercise was initially carried out at a moderate intensity, between 50 and 60% of HRM, progressing to 75–80% of HRM (Lochbaum & Crews, 2003; Fragala Pinkham et al., 2005). Strength training began at 60% of the 1 RM, increasing by approximately 5% per week until reaching 80–85% of the 1 RM (Lochbaum & Crews, 2003). Differences in objectives, available material resources and/or individual characteristics may explain the variation in training intensities used in the different studies, which did not always follow the usual ACSM recommendations (2022), which suggest working out at between 60 and 85% of the HRM for the aerobic component and 60–75% of the 1 RM for muscular strength. Although different intensities have been reported, in general, the ACSM (2022) guidelines were applied, and all of the studies reported positive effects (Table 3). The application of the training progression principle was a constant in several studies (Fragala Pinkham et al., 2005; Kozlowski et al., 2021; Lochbaum & Crews, 2003; Magnusson et al., 2012), with a gradual increase in intensity over the intervention period, regardless of the type of material or equipment used.

### Exercises

In this systematic review, the most common intervention was walking and/or running (Toscano et al., 2018; Yu & Jee, 2020), corroborating what the literature indicates; these exercises are often selected because they are accessible and adaptable to the different levels of functionality in ASD, making them more feasible for a broad spectrum of abilities (Bremer et al., 2016; Lang et al., 2010; Petrus et al., 2008; Sowa & Meulenbroek, 2012; Srinivasan et al., 2014; Tan et al., 2016). In addition, activities such as treadmill training, cycle ergometry, going up and down steps, obstacle courses, manipulation of objects and motor coordination exercises were carried out (Arslan et al., 2020; Bittner et al., 2021; Cha et al., 2020; Fragala Pinkham et al., 2005; Jimeno, 2019; Kozlowski et al., 2021; Lochbaum & Crews, 2003; Spratt et al., 2018; Toscano et al., 2018; Yu & Jee, 2020). With respect to strength training, the use of body weight, free weights, balls of different weights, resistance bands and shin guards in exercises such as plyometrics, step-ups, press-ups, curl-ups, climbing and barbell holds has been highlighted (Magnusson et al., 2012; Shields et al., 2018; Todd et al., 2019). The literature highlights several proposals for physical activities that have improved the motor proficiency of young people and adults with ASD. Among them, trampoline training (Lourenço, 2015) and aquatic activities, which have improved muscle strength, endurance, flexibility and cardiorespiratory fitness (Pan et al., 2011), are particularly recommended (Yanardag et al., 2014). Dance and core stabilization programs have also shown significant improvements in neuromuscular coordination and balance (Arzoglou et al., 2013; Golsefidi et al., 2013). Authors such as Okuda et al. (2014) emphasize the importance of sensory-motor, playful and kinesthetic activities, as well as stimuli aimed at spatial and temporal organization, body balance and fine motor coordination, for children with ASD.

### Evaluation Instruments/Techniques

VO^2^ Max was assessed using incremental, continuous and submaximal protocols on a treadmill (Modified Bruce) (Cha et al., 2020; Magnusson et al., 2012) or using functional tests such as the shuttle-run test (Fragala Pinkham et al., 2005; Yu & Jee, 2020). The assessment of neuromuscular capacity has been carried out mostly using the handgrip strength test (Cha et al., 2020; Yu & Jee, 2020), while the modified curl up (Cha et al., 2020) and push-up tests (Kozlowski et al., 2021) have also been used. According to the literature, submaximal effort protocols are a reliable way of assessing cardiorespiratory fitness in individuals with ASD (Ruivo, 2018). They are based on the linear relationship between VO^2^ and HR and are regularly used to observe the dynamics between exercise intensity and the responsiveness of the cardiovascular, pulmonary, musculoskeletal and neurological systems (Albouaini et al., 2007). The prescription of exercise intensity should be based on direct measurements of HRM, if possible, since an equation may not predict the true HRM in some individuals, specific populations or types of exercise (Tanaka et al., 2001). However, in young people with ASD, direct measurements are very difficult to use because of the behavioral characteristics of the population. For the results obtained to be positive, it may be necessary to apply adaptive procedures, such as in the research by Toscano et al. (2018): in the first session, the participant wore an 8 cm wide elastic band with an adjustable length across the chest for a minimum of 5 min; in the second session, he wore the same elastic band for a minimum of 15 min; in the third session, he wore the elastic band for the entire session; and in the fourth session, he wore the Polar RS800CX heart rate monitor for the entire session, recording the number of episodes of resistance while using the equipment.

The implementation of combined programs showed positive results in all studies, regardless of the different prescriptions, indicating that variables such as training frequency, exercise volume, load and repetitions can be adjusted to optimize the response to training. These findings are relevant to promoting the quality of life of individuals with ASD, in line with the conceptual model of Schalock and Verdugo (2002), which covers dimensions of (i) independence, (ii) social participation and (iii) well-being. The physical exercise recommendations, listed in Table 4, are based on the results of the combined programs reviewed.

**Table 4:** Recommendations for the prescription of physical exercise in individuals with ASD

Type of program	Objective	Training	Progression	Duration	Frequency	Volume	Intensity	Protocol	Evaluation test
Combined	Improving cardiorespiratory capacity	Aerobic	Continuous walking—continuous running—variable speed running	12 weeks	Minimum: 2 times a week Ideal: 3 times a week	Minimum: 45' Ideal: 60'	HR between 50 and 60% of HRM (Moderate Intensity), with the possibility of progressing to 75–80% (High Intensity)	(i) Shuttle-run Test Protocol (ii) Modified Bruce Protocol	(i) Shuttle-run Test (ii) Modified Bruce
	Improved muscular strength	Strength	Muscle endurance work -Resistant strength work		Minimum: 1 time per week Ideal: 2 times a week	Minimum: 10' Ideal: 20' 2–4 exercises 1–3 sets 6–12 repetitions (Moderate Intensity) 6–8 repetitions (High Intensity)	60–75% 1 RM (Moderate Intensity), with the possibility of progressing to 80% to 85% (High Intensity)	(i) Brockport Physical Fitness Test Manual	(i) Handgrip Strength Test (ii) Modified Curl Up (iii) Push Up Test

*'* minutes, *HR* heart rate, *MHR* maximum heart rate, *RM* repetition maximum

## Strengths and Limitations

This systematic review analyzed the effects of training on individuals with ASD, serving as a valuable reference for researchers and professionals in adapted sports. The studies reviewed offer guidelines and recommendations for the implementation of combined programs, with the aim of maintaining and improving physical fitness, quality of life and health, as well as reducing the risk of chronic diseases. This review highlights the importance of incorporating these programs into the weekly routine of this population, as when combined with a healthy lifestyle, they can provide various adaptations and benefits, including reducing healthcare costs, promoting healthy aging and improving general health.

Some studies included in this systematic review had limitations that may influence the magnitude of the results: (i) sex, age and level of functioning in ASD were not mentioned in some included studies; (ii) small sample sizes; (iii) samples composed exclusively of male individuals; (iv) unclear descriptions of the randomization process and allocation of participants with ASD to the training and control groups; (v) studies of short duration; (vi) diverse intervention methodologies involving different durations, frequencies, volumes, intensities and exercises; and (vii) different evaluation methodologies and results, making it difficult to conduct a more in-depth discussion and meta-analysis of the effects of the various combined programs applied. Future studies should consider these limitations when implementing combined programs. Therefore, an protocol study with a design that clearly specifies the characteristics of the exercise program, particularly in terms of managing and monitoring intensity, the adaptation procedures, the different phases of intervention and the type of evaluation used is needed to deepen the knowledge about exercise prescription in individuals with ASD.

## Conclusion

On the basis of the studies analyzed in this systematic review, interventions focused on cardiorespiratory fitness and muscle strength in individuals with ASD effectively improve cardiorespiratory capacity and hemodynamic parameters. The benefits of training are enhanced when both capacities are combined. However, the available evidence is insufficient to confirm significant improvements in body composition, especially lipid profiles and metabolic fitness, in individuals with ASD. In addition, the limited number of studies and the low methodological quality of some of them suggest a considerable risk of bias, which restricts the interpretation of the results and points to the need for more in-depth research. Thus, new studies on combined training programs are needed to better understand their characteristics and effects on individuals with ASD.

Of the 18 studies developed, the following aspects were considered transversal when prescribing combined programs: (i) Duration of 12 weeks for mesocycles; (ii) Most aerobic exercises are performed three times a week, while muscular strength exercises are performed twice a week; (iii) Each training session lasts 45 to 60 min for aerobic exercises and 10 to 20 min for muscular strength exercises; (iv) Two to four exercises are recommended that recruit the main muscle groups; (v) For each exercise, it is suggested to perform 1 to 3 sets with 6 to 12 repetitions (moderate intensity) or 6 to 8 repetitions (vigorous intensity); (vi) the intensity of aerobic exercises must be between 50 and 60% of the MHR (moderate intensity) and can progress up to 75% to 80% of the MHR (vigorous intensity). For muscular strength exercises, the recommended intensity is between 60 and 75% of the 1 RM (moderate intensity), with the possibility of progression to 80% to 85% of the 1 RM (vigorous intensity), but it is necessary an in-depth review of the intensity of the programs combined in the ASD, to increase the quality of information; (vii) Assessment protocols used in combined programs must be specific for people with ASD and implemented under conditions similar to those of the training regime. The most common assessment methods include the shuttle-run test protocol and the modified Bruce protocol. To assess muscle strength, the Brockport Physical Fitness Test Manual, which includes the manual grip strength test, the modified curl-up test and the push-up test, was used.
